# Development of a Method for Quantitative Evaluation of Facial Swelling in a Rat Model of Cerebral Ischemia by Facial Image Processing

**DOI:** 10.3389/fmed.2022.737662

**Published:** 2022-02-24

**Authors:** Yanfei Liu, Hui Huang, Yiwen Li, Jing Cui, Tiejun Tong, Hongjun Yang, Yue Liu

**Affiliations:** ^1^National Clinical Research Centre for Chinese Medicine Cardiology, Xiyuan Hospital of China Academy of Chinese Medical Sciences, Beijing, China; ^2^Second Department of Geriatrics, Xiyuan Hospital of China Academy of Chinese Medical Sciences, Beijing, China; ^3^Beijing Duan-Dian Pharmaceutical Research & Development Co., Ltd., Beijing, China; ^4^Department of Mathematics, Hong Kong Baptist University, Hong Kong, China; ^5^Medical Experimental Center, China Academy of Chinese Medical Sciences, Beijing, China

**Keywords:** ischemic stroke, occlusion of the middle cerebral artery, artificial intelligence, facial swelling, rat models

## Abstract

A quantitative method for the evaluation of facial swelling in rats with middle cerebral artery occlusion (MCAO) was established using a mathematical method for the first time. The rat model of MCAO was established *via* bilateral common carotid artery ligation. Three groups of rats with the same baseline were selected (model group, positive drug group, and control group) according to their behavioral score and body weight 24 h after surgery. Drug administration was initiated on post-MCAO day 8 and was continued for 28 days. Mobile phones were used to collect facial images at different time points after surgery. In facial image analysis, the outer canthi of both eyes were used as the facial dividing line, and the outer edge of the rat's face was framed using the marking method, and the framed part was regarded as the facial area (S) of the rats. The histogram created with Photoshop CS5 was used to measure the face area in pixels. The distance between the outer canthi of both eyes (Le) and vertical line from the tip of the nose to the line joining the eyes was recorded as H1, and the line from the tip of the nose to the midpoint of the line joining the eyes was recorded as H2. The facial area was calibrated based on the relationship between H1 and H2. The distance between the eyes was inversely proportional to the distance between the rats and mobile phone such that the face area was calibrated by unifying Le. The size of Le between the eyes was inversely proportional to the distance between the rats and mobile phone. This was used to calibrate the face area. When compared with the control group, the facial area of the model group gradually increased from postoperative day 1 to day 7, and there was a significant difference in the facial area of the model group on postoperative day 7. Hence, positive drugs exhibited the effect of improving facial swelling. H1 and H2 can reflect the state of turning the head and raising the head of the rats, respectively. Facial area was calibrated according to the relationship between H1 and H2, which had no obvious effect on the overall conclusion. Furthermore, mobile phone lens was used to capture the picture of rat face, and the distance between the eyes and H1 and H2 was used to calibrate the facial area. Hence, this method is convenient and can be used to evaluate subjective judgment of the human eyes *via* a quantitative method.

## Introduction

An important pathogenesis of ischemic stroke, which is a major disease that threatens human health, is the cascade of cerebral artery embolism and consequent inflammatory response (1, 2). The middle cerebral artery occlusion (MCAO) model is a clinically common simulation of ischemic stroke, which is less invasive and exhibits the closest resemblance to human ischemic stroke (3). Specifically, bilateral common carotid artery ligation and reperfusion is often employed in rats or mice to establish MCAO rat models for simulating the clinical features of ischemic stroke and performing pharmacodynamic evaluation. In the course of a routine rat MCAO model establishment and drug evaluation experiment, we determined that facial swelling occured in each group of rats after surgery, and the changes in facial swelling in each group exhibited certain characteristics as the duration of drug intervention increased. To avoid subjective evaluation via gross examination, we attempted to develop a convenient method for quantitatively evaluating facial swelling characteristics of the model rats. Furthermore, we employed some mathematical methods to maximally reduce the bias due to human manipulation to provide a multi-dimensional quantitative index for future pharmacodynamic evaluation (4, 5).

## Materials and Methods

### Materials

Thirty male specific-pathogen free (SPF)-grade 10-week-old Sprague-Dawley rats weighing 220–270 g were purchased from Beijing Vital River Laboratory Animal Technology Co., Ltd. (license number: SCXK (Beijing) 2016-0006) and housed in SPF-grade animal facilities. Donepezil hydrochloride tablets were purchased from Zhejiang Huahai Pharmaceutical Co., Ltd. (NMPA approval no. H20183417, lot number: 1426J20004). The positive drug used in this study was donepezil hydrochloride tablets, which was often used as a positive drug in vascular dementia and cerebral ischemia experiments (6).

### Methodology

#### MCAO Procedure and New Findings

**Figure 1:**
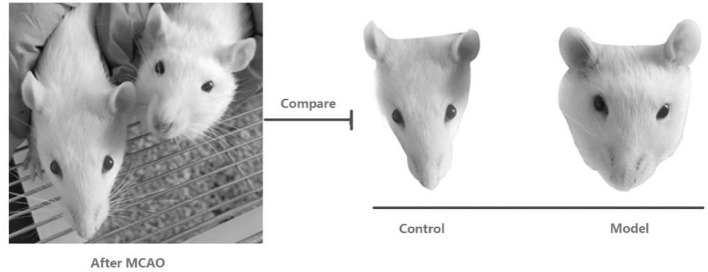
Comparative pictures of facial swelling after surgery in rats treated with middle cerebral artery occlusion.

The rats were anesthetized with 1 ml/100 g of 4% chloral hydrate via intraperitoneal injection and 1-cm incisions were made on the left and right regions of the neck. Blunt dissection of the superficial fascia was performed wherein the superficial fascia and intermuscular space among the digastric, sternocleidomastoid, and omohyoid muscles were separated. The bilateral common carotid arteries and vagus nerve were exposed. Furthermore, the common carotid artery and vagus nerve were carefully separated and two sutures were passed through the common carotid artery at the proximal and distal ends. The sutures were retained on the lateral side of the wound. The wound was sutured and ligation was maintained for 10 min, followed by 10 min of reperfusion. These steps were repeated three times. After the last reperfusion, the sutures were removed from the wound and the common carotid artery was permanently ligated with double sutures, and the right common carotid artery was ligated in the same manner as the left one. During the period from postoperative day 1 to day 35 at the end of the experiment, the model group showed significant facial swelling compared with the normal group that did not undergo surgery (Figure 1).

#### Grouping and Drug Administration

Twenty-four rats that underwent MCAO were bifactorally grouped according to behavioral scores and body weight after postoperative 24 h. After excluding rats with different baseline values, three groups of six rats, each with the same baseline values, were selected and classified into model group and positive drug group, and six rats that did not undergo MCAO were assigned to the control group. In the positive drug group, donepezil hydrochloride tablet was administered by gavage at a dose of 0.5 mg/(kg.d), and the model and control groups were provided equal volumes of distilled water via gavage daily. Administration was commenced on post-MCAO day 8 and was continued for 28 days.

#### Facial Image Acquisition and Analysis Process

**Figure 2:**
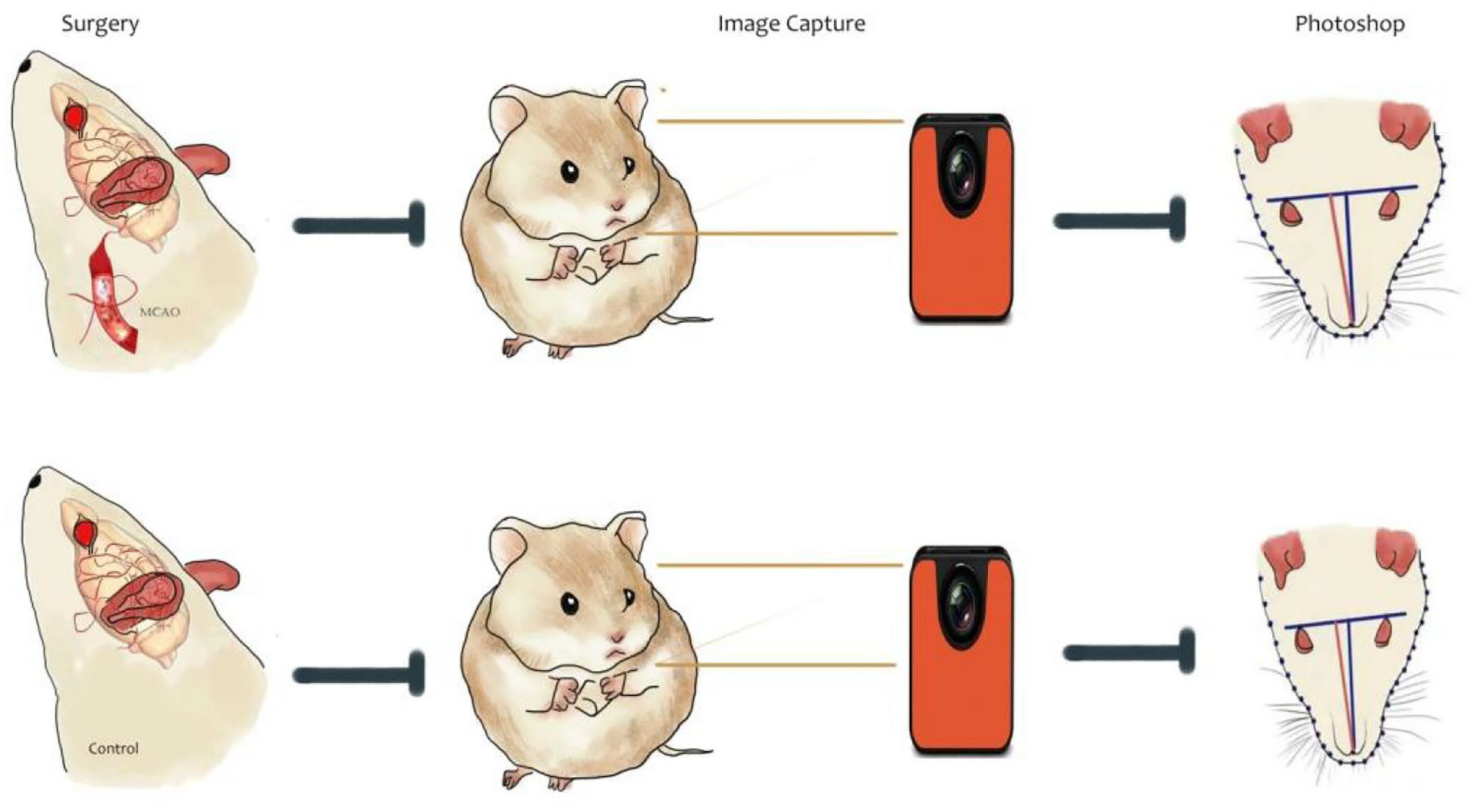
Schematic of the flow of facial image acquisition and analysis after cerebral ischemia in rats.

To further analyze the characteristics of facial swelling, we acquired facial images using a camera at different time points after each group of rats recovered autonomous behavior after performing MCAO. The experiment was divided into two stages. The first stage was from MCAO to the period before the administration of drugs. This stage lasted a total of 7 days, and facial images were collected on postoperative days 1, 3, 5, and 7. The second phase was from grouping and administration to the end of the experiment, and facial images were acquired on postoperative days 8, 12, 16, 20, 24, and 28. The drug administration was commenced on day 8 (Figure 2).

**Figure 3:**
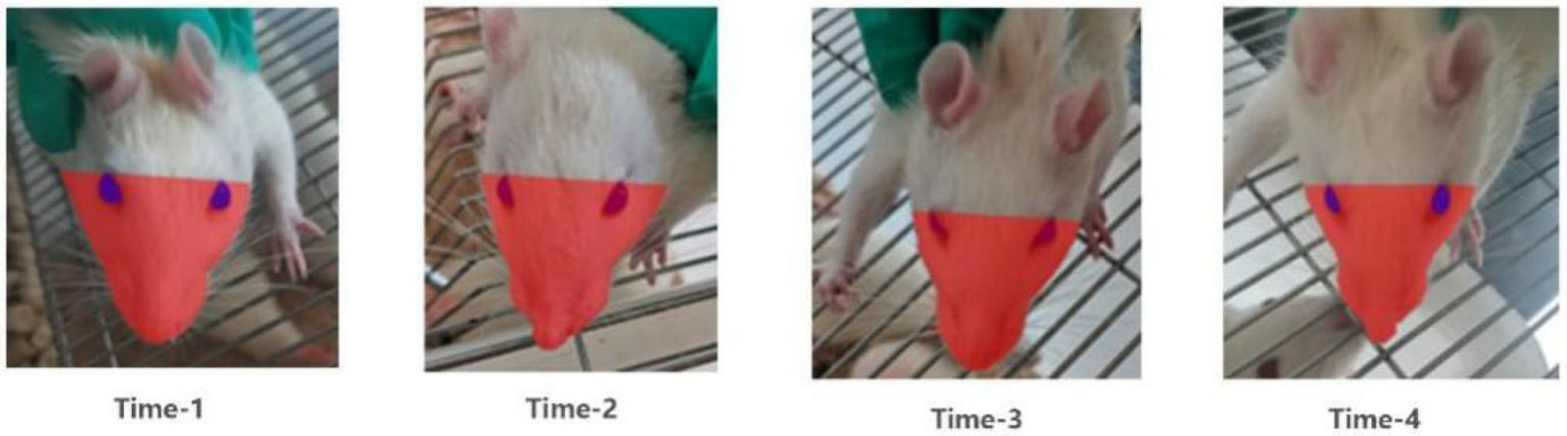
Images of facial recognition at different time points after surgery in the model group of rats.

The body and head of the rat were fixed with both hands during acquisition, and attempts were made such that the face of the rat faced the camera during photography. In particular, the head elevation angle and head rotation restriction were maintained as consistent to the maximum extent each time. Three images were acquired for each rat, and the image with the best angle and clarity was selected for calculation during analysis. The acquisition device was HUAWEI YAL-AL10, a mobile phone camera. The resolution of the camera at the time of photography was set as 72 × 72 DPI, and the image size was 3,000 × 4,000 pixels. The acquired images were imported into Photoshop CS5. The facial images were analyzed using the outer canthi of both eyes as the facial segmentation line. Furthermore, the outer edges of the face of the rat were boxed using markers and the boxed portion was considered as the total facial area (facial area, S) of the rats. Finally, the facial area was measured in pixels using the histogram in Photoshop CS5 (Figure 3). The distance between the outer canthi of both eyes (Le) and vertical line from the tip of the nose to the line joining the eyes was recorded as H1, and the line from the tip of the nose to the midpoint of the line joining the eyes was recorded as H2. The length was measured in pixels using the histogram tool in the software (4, 5). Thus, by following this method (4, 5), we acquired facial images of each group of rats at each postoperative time point (Figure 3).

### Facial Image Calibration Methods

**Figure 4:**
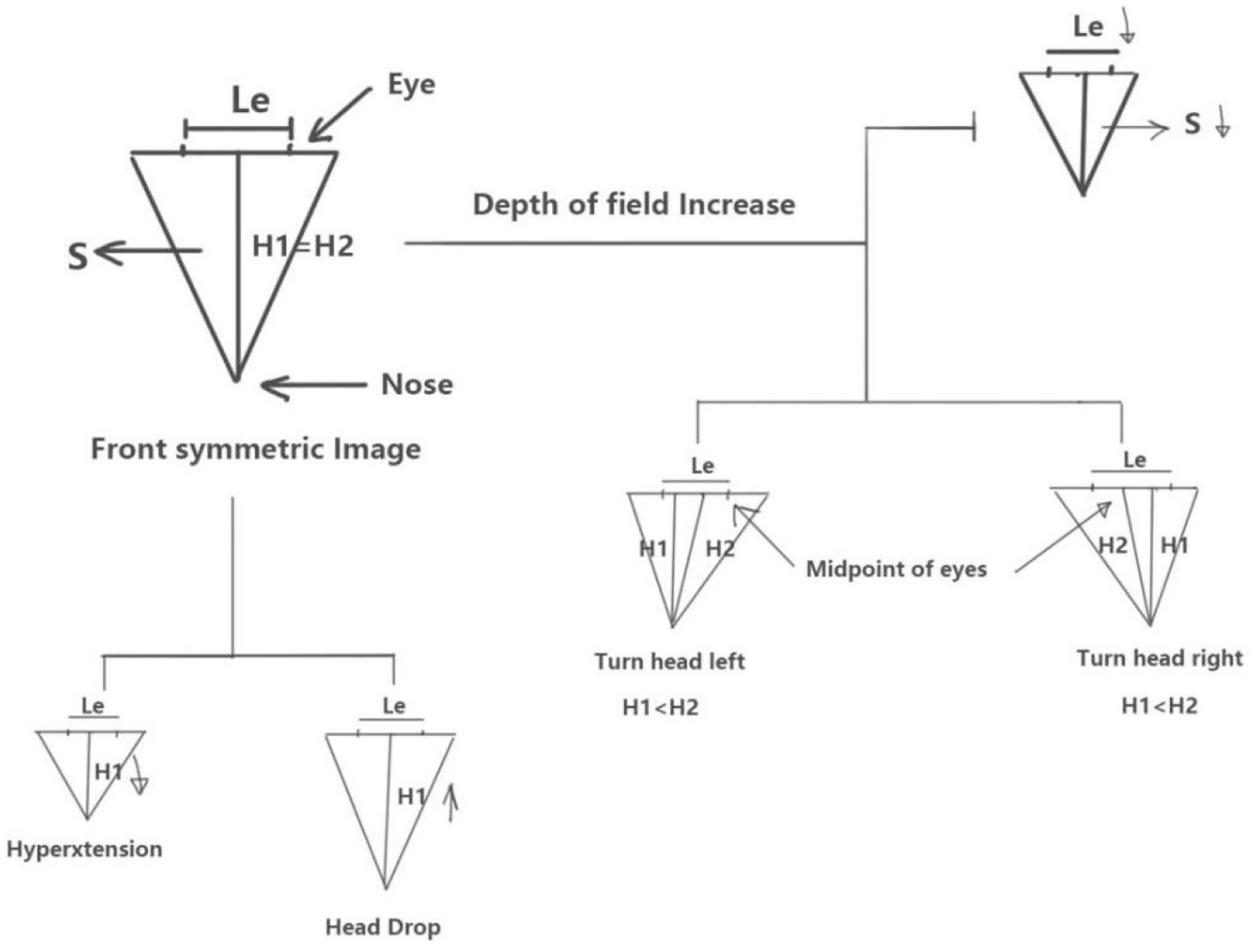
Schematic of the calibration method using mathematical interpretation of head–facial variables.

We didn't anesthetize the rats or install any assembly equipment to make the whole process as easy as possible. Therefore, in the process of taking photos, there would be uncontrollable factors such as the distance between the lens and the target and the head swing of rats in different directions, but we found that these problems can be corrected by simple mathematical methods. During the facial image acquisition process, we determined that the distance of the camera lens from the target object, the lifting or lowering of the head of the rat, and frontal and head-turned images affected the facial image acquisition and results. To minimize this interference, we utilized a simple mathematical principle (4, 5) for calibration (Figure 4). When the lens is turned away from the face of the rat, the distance Le between the outer canthi of the eyes decreases and point S decreases accordingly. When the face of the rat is lifted, H1 shortens and S decreases, while Le is assumed to be constant. Conversely, when the head of the rat is lowered, H1 and S increase while Le is assumed as constant. When the rat is facing the lens, H1 = H2, and when the rat turns its head, either to the left or to the right, H2 > H1. When this occurs, the facial area S of the rat also appears to increase or decrease with respect to H1 and H2, while Le is assumed as constant.

**Figure 5:**
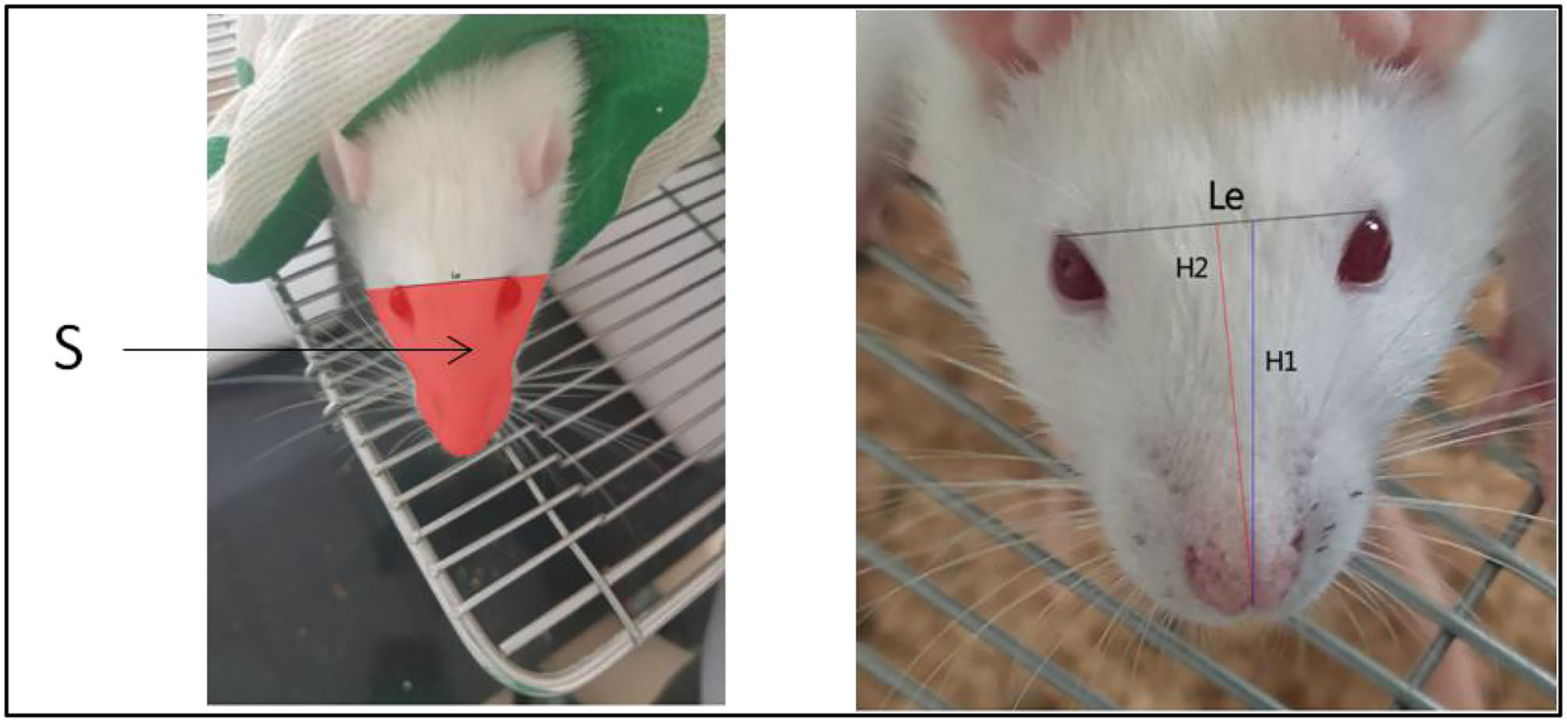
Schematic of each parameter of rat face. Red denotes the area of the face recognized (S).

As described above, the vertical length H1 from the tip of the nose to the line connecting the two eyes is indicated by the blue line, and the length H2 from the midpoint between the two eyes to the tip of the nose is indicated by the red line as shown in Figure 5.

### Statistical Analysis

Statistical analysis was performed with SPSS (version 22.0). All data were expressed as mean ± SD. The comparisons between multiple groups were analyzed by one-way ANOVA, and group comparisons were analyzed using Student's *t* test. A *P* < 0.05 was considered statistically significant.

## Results

### Calibration of the Distance Between the Lens and Target Object

As the distance between the camera and subject decreases, the subject's face area increases. Conversely, as the distance between the camera and object increases, the face area decreases. To ensure that the technique is adaptable for use in experiments, the operator did not fix the distance between the lens and target when capturing. Hence, Le, H1, and S decreased as the lens moved away from the target. Specifically, in this case, we considered the basic principles of digital and aesthetic anatomy wherein the interocular distance is determined by the brow bone and is fixed for the same types of rats. Therefore, we can determine the distance of the lens from the target object while taking a picture based on the size of the Le of the same type of rats. When the lens is turned away from the face of the rat, the distance Le between the outer canthi of the eyes decreases and point S decreases accordingly. Similarly, we can normalize Le of the same type of rats and use it to convert the area under the same Le to eliminate the changes in the absolute value of facial area due to the varying distance of the lens.

**Figure 6:**
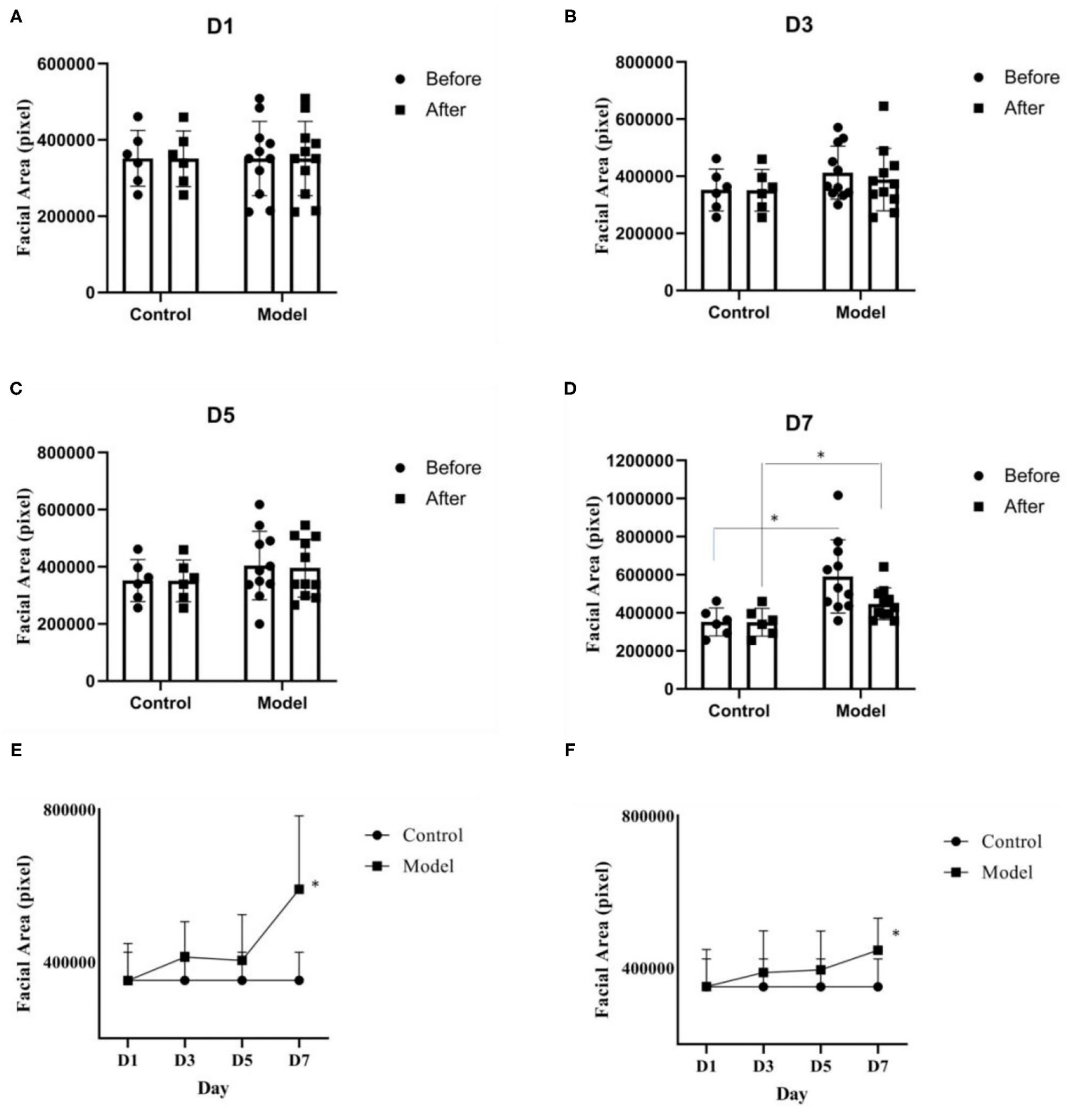
Comparison of parameter S in each group before and after Le calibration in the postoperative to pre-dosing phase. Comparison of S values in each group before and after Le calibration: **(A)** on day 1 after surgery, **(B)** on day 3 after surgery, **(C)** on day 5 after surgery, **(D)** on day 7 after surgery. Curves of changes in S values at different time points in the two groups: **(E)** before calibration and **(F)** after calibration. Model group compared with control group, *p < 0.05. control group: *n* = 6, model group: *n* = 11.

As described in Figure 6, we normalized the interocular Le of the same rats at different time points and converted the facial area of the rats as described above. The results indicated that there was no significant change in trend between the groups at different time points after calibration compared with that before calibration (Figures 6A–D). However, the absolute value of facial area changed, which is also consistent with our description above. After performing MCAO, the model group showed an increase in facial area from postoperative day 3 compared with the control group. Furthermore, a significant difference (*p* < 0.05) was observed on postoperative day 7 as the postoperative duration increased (Figures 6E,F).

**Figure 7:**
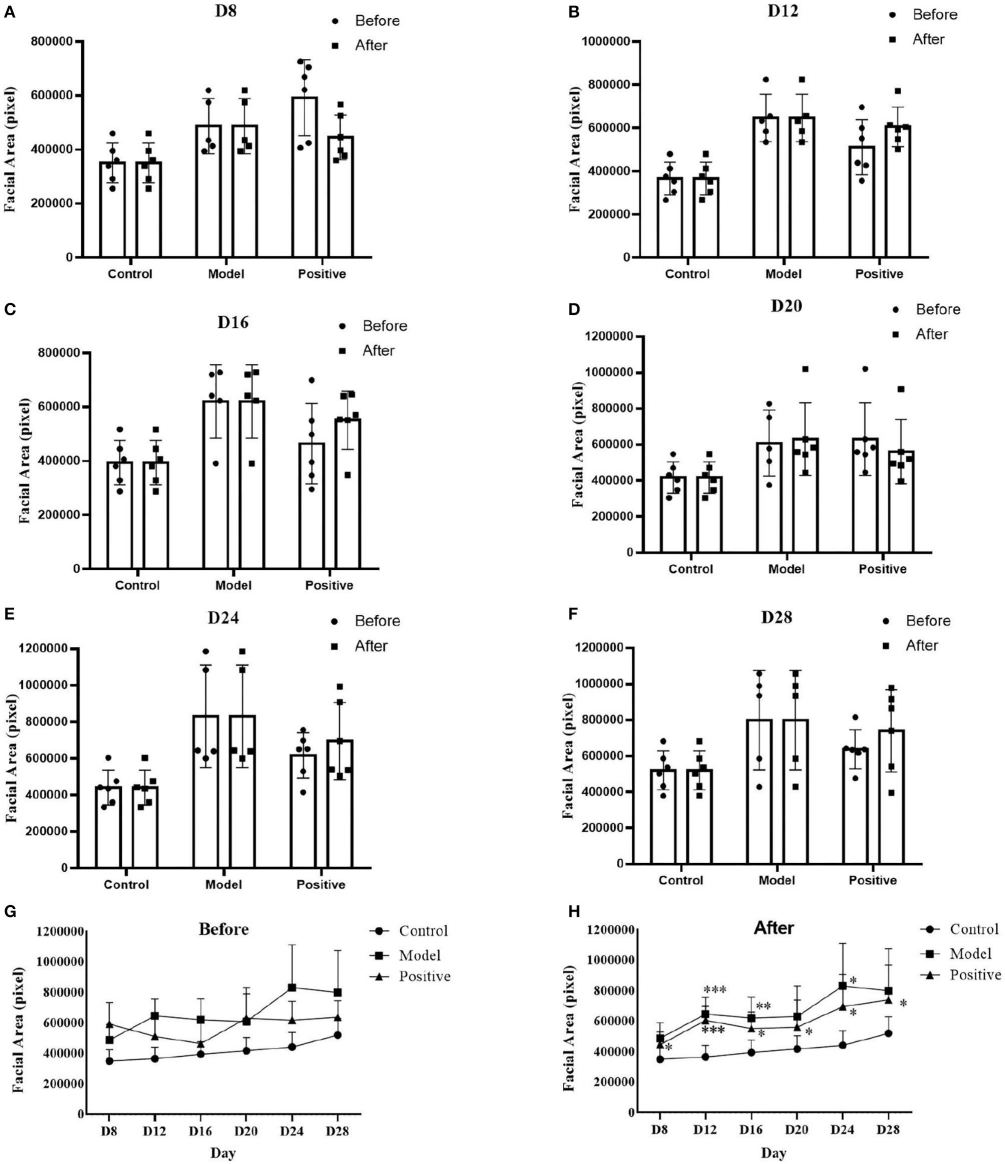
Comparison of parameter S before and after Le calibration in each group after dosing to the end of the experiment. Comparison of S values before and after Le calibration: **(A)** on experiment day 8 (day 1 after dosing), **(B)** on experiment day 12 (day 5 after dosing), **(C)** on experiment day 16 (day 9 after dosing), **(D)** on experiment day 20 (day 13 after dosing), **(E)** on experiment day 24 (day 17 after dosing), **(F)** on experiment day 28 (day 21 after dosing); S value change curves of different groups, **(G)** before Le calibration, and **(H)** after Le calibration. Compared with the normal group, *p < 0.05; **p < 0.01; ***p < 0.001. Model group: *n* = 5, control group: *n* = 6, positive drug group: *n* = 6.

The experimental results in Figure 7 indicate that the S values of the different groups during the administration differ before and after Le calibration. On day 1 after dosing (day 8 after performing MCAO), the model group showed a significant increase in facial area compared with the normal group (*p* < 0.05). On day 5 after administration (day 12 after performing MCAO), the model and positive drug groups exhibited a significant increase in facial area compared with the normal group (*p* < 0.001). However, the active control group exhibited a lower facial area than the model group. From day 9 (day 16 after MCAO) to day 21 (day 28 after MCAO), the facial area of the model group was higher than that of the normal group. This indicates that bilateral common carotid artery ligation during MCAO can lead to facial swelling in rats. This can be relieved after the administration to the positive drug group.

### Calibration of Head Rotation

**Figure 8:**
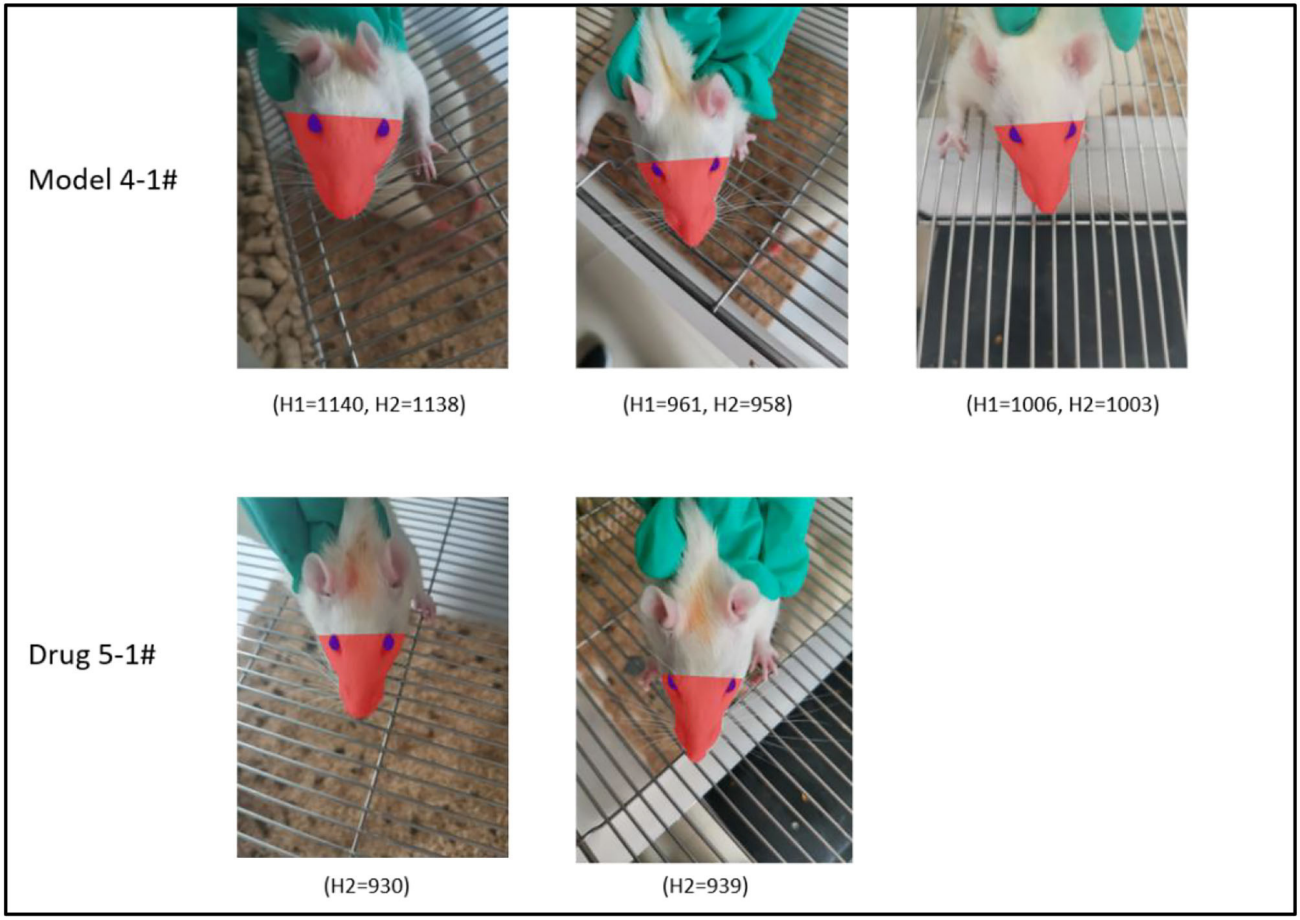
Demonstration of the head angle calibration effect. Row 1 shows rat #4 in the model group, H1 and H2 are close at all time points, and the face of the rat faces the front in all cases. Row 2 shows rat #5 from the investigational drug group, H2 is close at various time points, and the head angle is approximate.

When grasping rats, head rotation often occurs. In this case, we considered the occurrence of head rotation when H1 < H2 according to Figure 4. Figure 8 shows pictures of model group No. 4 rat acquired at different time points. The H1 value of the rat is very close its H2 value. The pictures in first line of Figure 4 indicate that the lower part of the rats is more symmetrical at each time point. Furthermore, we considered that the No. 4 rat's face was facing the camera lens in this case.

**Figure 9:**
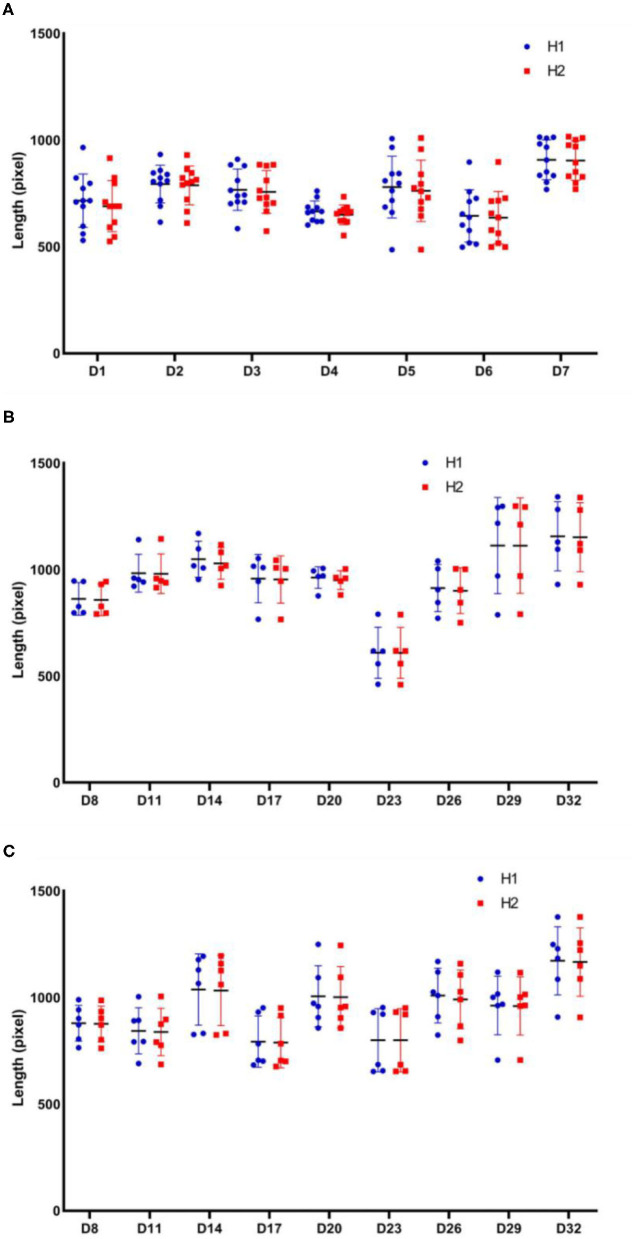
Comparison of H1 and H2 parameters for each group at different stages of the experiment. H1 and H2 dynamics curves for each rat: **(A)** in the model group after surgery and before administration; **(B)** in the model group between the beginning of administration and end of the experiment; **(C)** in the active control group between the beginning of administration and end of the experiment. Model group: *n* = 5, positive drug group: *n* = 6.

We measured H1 and H2 parameters for each rat in the model group after surgery until drug administration. By acquiring pictures at successive time points, we observed that the mean values of H1 and H2 did not differ significantly at each time point (Figure 9A). Therefore, we concluded that the calibration of H1 and H2 was weaker than that of Le described above. To further assess the effect of this parameter on facial recognition, we measured H1 and H2 from the model and active control groups at each time point between the administration of the drug and end of the experiment. A comparison of the results indicate that H1 and H2 were identical (Figures 9B,C).

### Lifted Head vs. Lowered Head Calibration

The distance between the tip of the nose and line between the eyes decreased when the head was tilted upward. Conversely, the distance between the tip of the nose and line between the eyes increased when the head was tilted downward. During this time, the distance between the eyes remained constant (assuming the lens was at the same distance from the target) (Figure 8). As shown in Figure 8, rat #5 in the active control group exhibited similar H2 values at the two different time points, and the angle of head elevation was also very close.

**Figure 10:**
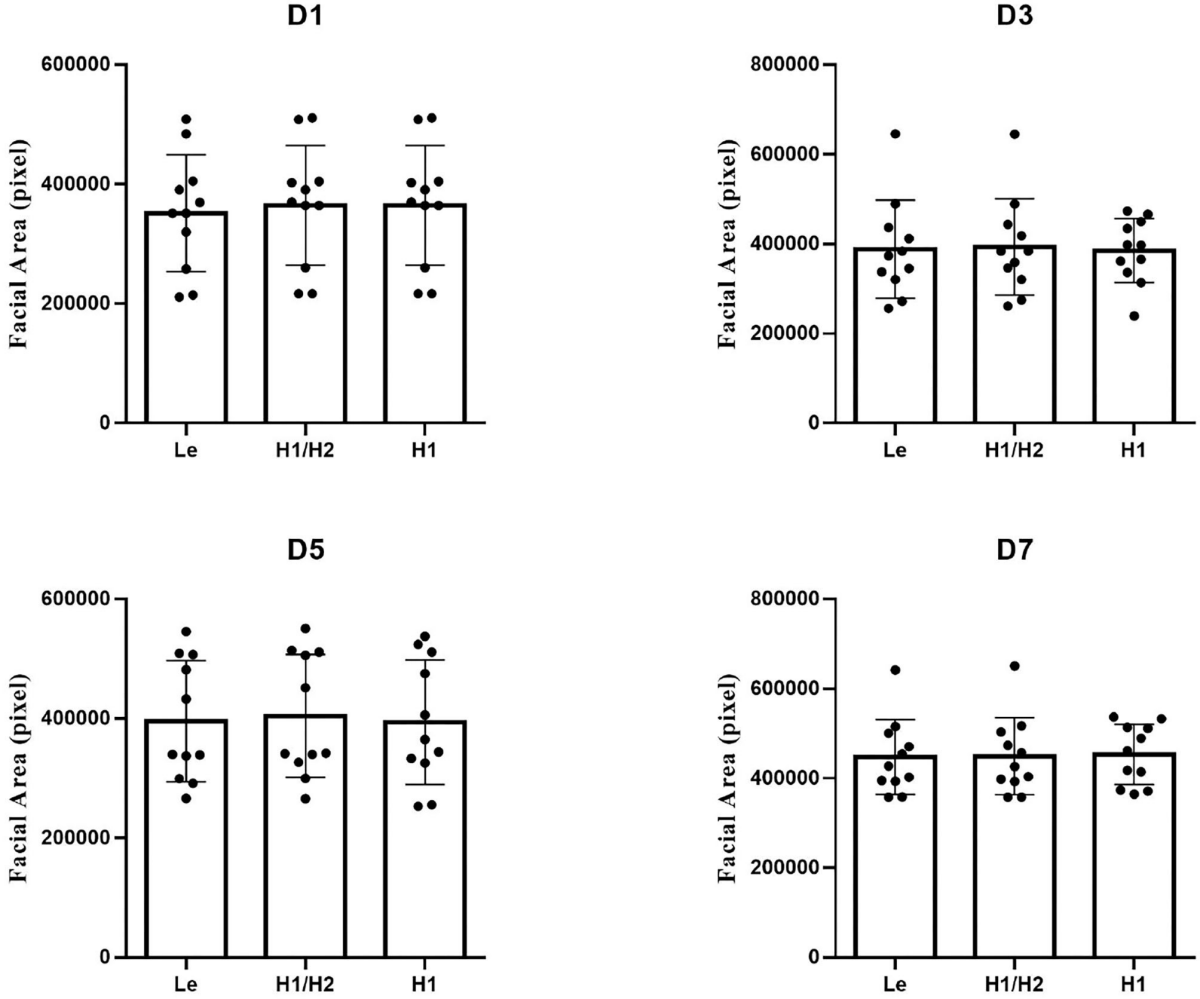
Effect of different calibration methods on S in the model group in the postoperative to pre-dosing phase. Model group: *n* = 5.

We calibrated the facial area of each rat in the model group from the postoperative to the pre-dosing phase in the order of mirror depth (Le), head rotation (H1 vs. H2), and head lifting (H1). Specifically, first, Le was calibrated based on the uncalibrated facial area (S) to obtain the calibrated area (leftmost bar of each part of Figure 10). Then, S data were calibrated according to the relationship between H1 and H2, and each rat was calibrated such that the area of each rat corresponded to the area when it was facing the camera (middle bar of each figure in Figure 10). Finally, based on S calibrated in the second step, H1 of all rats was normalized to obtain the final calibrated facial area of rats (H1 in each part of Figure 10). The results indicate that the deviation of S for each rat in the group decreased as calibration was progressively performed, thereby indicating that the calibration led to further representation of the objective facial area of the rat.

## Discussion

In this study, during the process of MCAO model construction and subsequent drug evaluation, we unintentionally determined that the model rats exhibited facial swelling compared with the control rats. To objectively evaluate the degree of facial swelling in rats, we used the most common photography method (mobile phone photography), which is flexible and can be performed at any time during the experiment. After image acquisition, we used Photoshop, common commercially available image processing software, for parameter measurements.

Anatomy is the basis of medicine and biology. With advances in technology, computer techniques have been increasingly applied to anatomy (7) such as digital pathology, image digitization, and three-dimensional scanning of the head. Furthermore, computer techniques are applied in plastic surgery and treatment of vascular diseases in the maxillofacial region (8). To enhance the representation of facial swelling features, we referred to anatomical and plastic surgery-related concepts to compute and analyze acquired images *via* custom parameter settings and calibration principles. Regarding the acquired images of the variable rat faces, we utilized the anatomy of the skull to capture the invariant brow bone of the same rats and used the distance between the eyes as one of the calibration methods to eliminate differences due to the distance between the lens and target. Simultaneously, we also adopted basic mathematical principles to determine the deviation due to the head tilting or head rotation of rats to perform a simple quantitative analysis. By performing a series of calibration analyses and comparisons, we concluded that the size of S was most closely related to the depth of the lens. Furthermore, as the distance between the rat and lens decreased, the interocular distance Le between the eyes increased and S increased at that time. Therefore, Le calibration is extremely critical in data analysis. Conversely, the calibration of H1 and H2 slightly affects the change in the size of S.

A literature search was conducted to address the biological explanation of the phenomenon of facial swelling after MCAO (9). However, to the best of our knowledge, there are no reports on the phenomenon of facial swelling in rats after MCAO. Several studies reported that shoulder-hand syndrome occurs after stroke, with one of its typical features corresponding to hand swelling. It has also been reported that stroke patients tend to exhibit deep vein thrombosis (10), and one of its classical clinical signs involves swelling of the affected limb. Based on these results, we hypothesized that facial swelling can occur in patients with cerebral ischemia. After reviewing the literature and based on our previous research experience, we believe that the causes of facial swelling after cerebral ischemia are as follows: (1) a sharp decrease in cerebral blood flow due to bilateral common carotid artery ligation (11), which in turn increases intracranial pressure. Some clinical trials have shown that inadequate venous drainage triggered by bilateral radical neck dissection can cause intracranial hypertension, which leads to facial swelling. It is hypothesized that carotid artery ligation affects venous return. This in turn results in facial swelling. (2) Bilateral common carotid artery ligation can lead to cell swelling and tissue edema (12), and patients with hypoxic–ischemic brain damage are more likely to exhibit cerebral edema (13). Additionally, acute intracranial pressure elevation (14) can cause periventricular leukomalacia. (3) Clinically, the middle cerebral artery (MCA) trunk exhibits a higher chance of stenosis or even occlusion than the anterior and posterior cerebral arteries (15). This is mainly because the MCA trunk has a higher blood flow and is more prone to atherosclerotic plaques and mural thrombi. Hence, this results in luminal narrowing (16). Conversely, occlusion of the superior cortical branch of the MCA can lead to contralateral involvement and impaired circulation (17). (4) The craniofacial and temporal fascia contain rich blood supply (18), which is derived from the common carotid artery, superficial temporal artery, facial artery, and maxillary artery, which are accompanied by veins and intertwined into a network at the terminal branches of the internal carotid artery. Therefore, some patients with severe stenosis of the extracranial segment of the internal carotid artery (more than 70% stenosis) can be treated by mandibular carotid endarterectomy (19, 20). The swelling of the maxillofacial region, which is observed using contrast techniques, is associated with compensatory thickening of the facial arteries (21). (5) Swelling of the maxillofacial region is closely associated with the onset of inflammation. Furthermore, facial swelling is observed in chronic angioneurotic edema (22), which is mainly due to capillary dilation, congestion, and exudation in deep connective tissue, and it is accompanied by inflammatory cell infiltration (23). Conversely, the tissues of the eyelids, upper and lower lips, and cheeks are relatively loose and are easily observed when edema occurs.

The positive drug used in this study was donepezil hydrochloride tablets. They are routinely used in clinical practice and can reversibly inhibit acetylcholine hydrolysis by acetylcholinesterase, thereby increasing the concentration of acetylcholine and exerting therapeutic effects by enhancing the function of cholinergic nerves. During the 28-day period of control administration to MCAO-treated rats, we observed that the drug had some ameliorative effect on facial swelling in the model rats after surgery. However, its effector mechanism is not known.

In addition, we analyzed the ocular characteristics of rats in the acquired images, including the eye area and proportion of the face occupied by the eyes (data not shown in this paper) and observed that the eyes of rats can protrude early and atrophy later after MCAO. This is also related to the fact that the blood supply to the eyes mainly comes from the branches of the internal carotid artery and cases of exophthalmos in stroke patients have been reported. Bilateral common carotid artery ligation leads to an increase in intraocular pressure of the body, which results in protrusion and atrophy of the eyes.

In summary, in this study, we established a simple and easy method to significantly replace the existing subjective scoring methods for edema and provide new ideas for future applications based on the analysis of facial swelling in stroke patients.

## Data Availability Statement

The original contributions presented in the study are included in the article/supplementary material, further inquiries can be directed to the corresponding authors.

## Ethics Statement

The animal study was reviewed and approved by Xiyuan Hospital of China Academy of Chinese Medical Sciences.

## Author Contributions

YuL and HY contributed to the design of experiments, manuscript revision, and decisions involving submission for publication and co-corresponding authors. YaL and HH performed the experiments and prepared the manuscript. YiL, JC, and TT analyzed the data. All authors edited the manuscript and provided final approval for the final version for publication.

## Funding

This study was supported by the Special Project for Outstanding Young Talents of China Academy of Chinese Medical Sciences (ZZ15-YQ-017 and ZZ13-YQ-001-A1).

## Conflict of Interest

HH was employed by the company Beijing Duan-Dian Pharmaceutical Research & Development Co., Ltd., Beijing, China. The remaining authors declare that the research was conducted in the absence of any commercial or financial relationships that could be construed as a potential conflict of interest.

## Publisher's Note

All claims expressed in this article are solely those of the authors and do not necessarily represent those of their affiliated organizations, or those of the publisher, the editors and the reviewers. Any product that may be evaluated in this article, or claim that may be made by its manufacturer, is not guaranteed or endorsed by the publisher.
